# A Computer-Aided Detection System for Digital Chest Radiographs

**DOI:** 10.1155/2016/8208923

**Published:** 2016-05-31

**Authors:** Juan Manuel Carrillo-de-Gea, Ginés García-Mateos, José Luis Fernández-Alemán, José Luis Hernández-Hernández

**Affiliations:** ^1^Computer Science and Systems Department, Faculty of Computer Science, University of Murcia, 30100 Murcia, Spain; ^2^Academic Unit of Engineering, Autonomous University of Guerrero, 39087 Chilpancingo, GRO, Mexico

## Abstract

Computer-aided detection systems aim at the automatic detection of diseases using different medical imaging modalities. In this paper, a novel approach to detecting normality/pathology in digital chest radiographs is proposed. The problem tackled is complicated since it is not focused on particular diseases but anything that differs from what is considered as normality. First, the areas of interest of the chest are found using template matching on the images. Then, a texture descriptor called local binary patterns (LBP) is computed for those areas. After that, LBP histograms are applied in a classifier algorithm, which produces the final normality/pathology decision. Our experimental results show the feasibility of the proposal, with success rates above 87% in the best cases. Moreover, our technique is able to locate the possible areas of pathology in nonnormal radiographs. Strengths and limitations of the proposed approach are described in the Conclusions.

## 1. Introduction

Medical imaging is a key field in healthcare engineering, which aims to help medical professionals to identify lesions and diseases. Early attempts at computerized analysis of medical images were made in the 1960s, such as diagnosis of primary bone tumor [1] and detection of abnormalities in mammograms [2]. In the 1980s a new concept emerged,* computer-aided diagnosis* (CAD) which assumed that the computer output could be utilized to assist physicians, but not to replace them. Currently, CAD systems are employed in the early detection of pathologies, that is, to obtain a “second opinion” and help them make the final decision [3–6]. A CAD system can also be very useful to provide some basic information when the human expert monitoring is not possible.

Each biomedical image technique is appropriate for certain diagnostics. For example, MRI enables the spatial localisation required for cross-sectional imaging whereas ultrasound images allow physicians the visualisation of soft tissues and have revolutionised obstetric care [7]. However, digital radiology is still the backbone of diagnostic bioimaging, mainly due to three reasons: (1) its capability to detect unsuspected pathologies; (2) being not invasive; and (3) having a low radiation dose and low cost [8].

**Figure 1:**
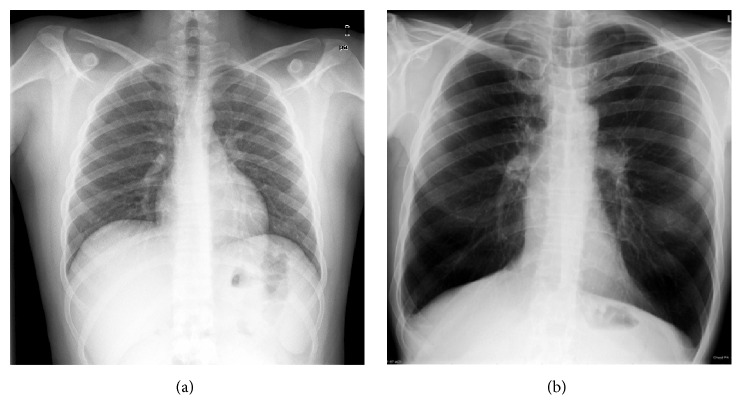
Sample chest radiographs in posteroanterior view. (a) Normal. (b) Pathological.

The majority of the studies related to CAD research have been concerned with some organs such as chest, breast, colon, and liver [5, 9–11]. The objective of this paper is to perform an automatic normality/pathology classification of posteroanterior (PA) digital chest radiographs. The proposed method is not specialized in a given set of types of lesions or diseases but is able to detect anything that differs from normality. A sample view of the radiographs under study is shown in Figure 1.

Although there is much computer vision research in CAD techniques, the problem studied here has received little attention so far. For example, we can cite some interesting research on CAD systems that work with mammography for breast nodule detection [12, 13]. Also, there are examples of systems focused on lung nodule detection using computer tomography [14, 15] or radiography [16–18]. These research efforts have resulted in commercial systems available in clinical practice [19].

Besides, some authors have proposed CAD systems capable of recognizing diseases such as polyps in the colon [20], acute intracranial haemorrhage [21], and severe respiratory syndrome [22]. Due to its importance in CAD systems, much research work has been devoted to the segmentation of anatomical regions of the body. Related to thoracic medical imaging, attention is directed particularly to the lungs [8, 23, 24], the lung fields, the heart and the clavicles [25], certain lung structures as hilar region [26], and the liver and neighboring abdominal organs [10, 11]; the latter two methods do not use simple radiographs, but other 3D image modalities such as CT and MRI. Some authors are also investigating how to segment the bony structures of the chest [27, 28], often to eliminate the shadows projected on the lung parenchyma.

On the other hand, much less research has been dedicated to the generic problem of discriminating normality from pathology. In this field, we can find the approach described in [29], which tackles the classification normal/nonnormal of radiographies of the chest. A *k*-nearest neighbors (*k*-NN) classifier is proposed using as input features the responses to a set of Gabor wavelet filters. Another interesting work is [30] that used computed tomographies (CT) for the problem of lung texture recognition. They used a LBP operator extended to 3D, performing a comparison of LBP histograms. These authors also presented a texture classification-based system for emphysema quantification in CT images comprising three classes: normal tissue, centrilobular emphysema, and paraseptal emphysema [31]. The present paper is an extension of the preliminary work described in [32], with a substantial improvement in the proposed method and the experimental validation.

## 2. Materials and Methods

A sample of 48 high resolution DICOM images of chest radiographs (25 males and 23 females) were provided by the Hospital General Universitario Reina Sofia de Murcia (HGURSM), Spain, to perform tests. The local Ethics Committees of the HGURSM approved the study, and written informed consent was obtained from the radiologist in charge of the diagnostic procedure at HGURSM. The images have a resolution of 3000 × 3000 pixels and a depth of 12 bits per pixel. In the available images, there are 25 normal (12 males and 13 females) and 23 pathologic (13 males and 10 females) samples. The ages of the subjects range from 15 to 93 years, with an average of 55.

**Figure 2:**
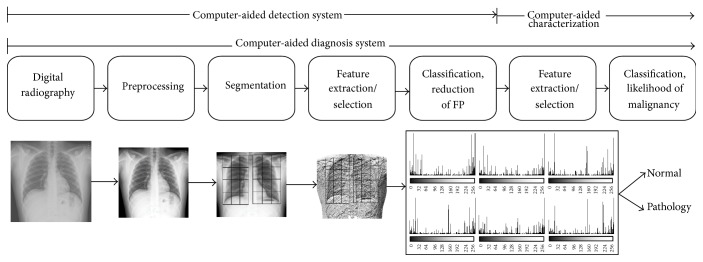
Typical scheme of a CAD system as proposed by [33]. Below each generic step, a sample image of the proposed method is shown.

The proposed image classification method is described in the following subsections. A global view of the developed system is shown in Figure 2.

### 2.1. Preprocessing and Segmentation

The first stage of the system is preprocessing and segmentation. In this step, the input DICOM files are reduced in pixel depth, from 12 to 8 bits per pixel. After that, decimation is applied to the images using supersampling interpolation, reducing the size to 1000 × 1000 pixels, that is, the standard resolution for the following steps.

In general, segmentation procedures are used to identify regions containing certain kinds of lesions [34]. In our system, the image is segmented to locate the position of both lungs in the radiographs, in order to determine the areas of interest. The proposed segmentation method is based on the template matching algorithm [35], which is a well-known technique in computer vision. This process consists in searching for a given template in all possible locations of an image, applying a predefined similarity measure for each location.

**Figure 3:**
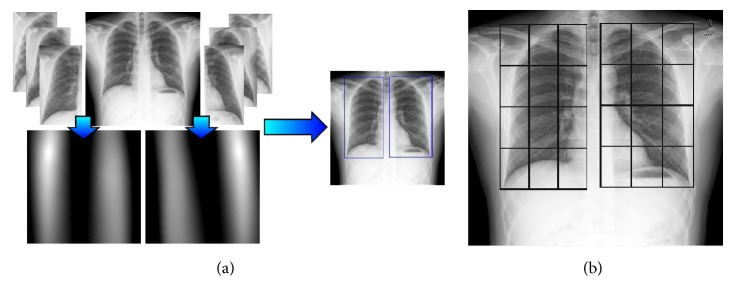
Lung location and segmentation in a radiograph. (a) Application of template matching to a radiography (left and right patterns), matching maps, and the obtained optimal location. (b) The detected lungs are divided into two grids of regions.

Samples of right and left lungs, extracted from the training set, are used as templates in the matching process. Different patterns of lungs are used to cope with the variety of aspects they can adopt due to sex, age, or individuals. The value applied in the matching algorithm is a correlation coefficient [36], which produces normalized values near 1 for the optimal location of the matching. Therefore, the location with maximum correlation is selected as the expected position of each lung. Afterwards, left and right lungs are segmented in square grids of 3 × 4 regions, as depicted in Figure 3. Observe that the proposed method does not produce a precise segmentation of the lungs contour, but a bounding box for each lung, which is sufficient for the subsequent processes.

### 2.2. Feature Extraction with LBP

**Figure 4:**
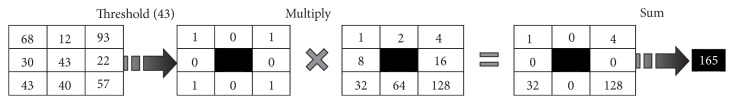
Example of a LBP calculation.

The aim of this step is to produce meaningful texture descriptors for the regions of interest. Different kinds of features have been used for biomedical images such as Fourier transform, wavelet filters, and SIFT features. The technique proposed in this paper is based on LBP features, which were introduced in [37]. LBP are an invariant texture descriptor that produces a value for each pixel in the images. Let us consider a single channel image, *I*, with an arbitrary photometric resolution. The LBP computation for a pixel *I*(*x*, *y*) takes into account the 8 pixels surrounding point (*x*, *y*), using the following equation:

(1) LBPx,y=∑n=072ntIx,y−Ineighn,x,y,with  tv=0,v≥01,v<0,

where neigh iterates the neighbors of pixel (*x*, *y*), that is, {(*x* − 1, *y* − 1), (*x*, *y* − 1), (*x* + 1, *y* − 1), (*x* − 1, *y*), (*x* + 1, *y*), (*x* − 1, *y* + 1), (*x*, *y* + 1), (*x* + 1, *y* + 1)}, and *t*(*v*) is a function that thresholds its parameter *v*.  Figure 4 shows a graphical representation of the computation of the LBP for a single pixel.

Each LBP(*x*, *y*) can take 256 values, from 0 to 255, encoding gray-level information with respect to the central pixel (*x*, *y*). These values are not taken individually; instead, they are aggregated in histograms for each region of interest. Given a region *R*, which consists of a set of pixels, the corresponding histogram *H*_*R*_ is given by

**Figure 5:**
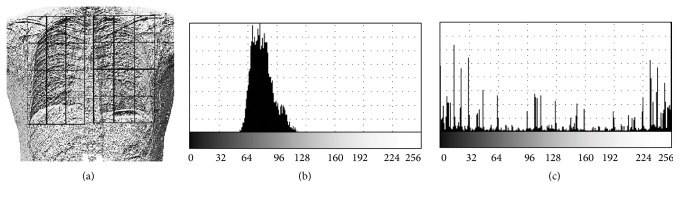
Sample application of LBP to a radiography. (a) LBP image of the radiography in Figure 3. (b) Histogram of gray levels of the original image. (c) LBP histogram for the same region.

(2) HRi=1R∑p∈Reqi,LBPp,with  eqa,b=1,a=b0,a≠b,

where *i* goes from 0 to 255. Observe that the histograms are normalized dividing the result by |*R*|, that is, the size of the region in pixels. A sample application of LBP histograms is shown in Figure 5, as compared to the histogram of gray levels of the original radiography. Note that all bits of the LBP image contain relevant information, but this may not be clearly seen in the image (only the most significant bits are appreciated in a visual inspection by humans).

**Figure 6:**
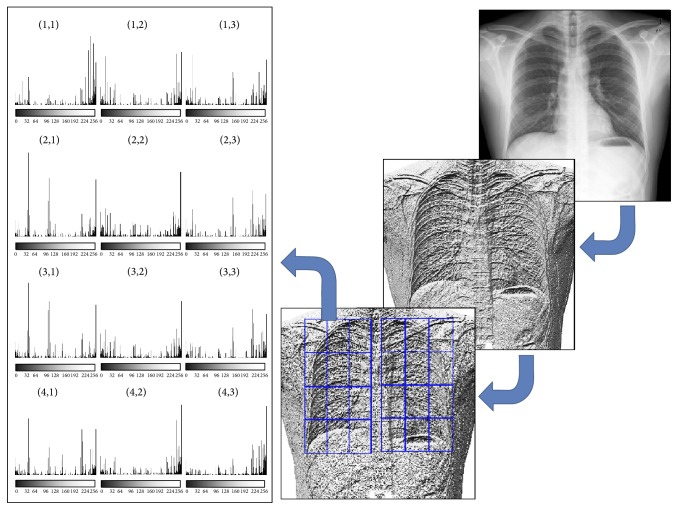
Calculation of LBP histograms in a sample chest radiograph. From right to left: input radiograph; computed LBP image; segmented regions; and LBP histograms obtained for each region of the left lung.

As mentioned before, the input radiography is divided into a grid of 3 × 4 regions for both lungs, which are determined according to the segmentation step. The LBP histogram of each region is obtained, producing a feature vector of 24 histograms of 256 bins.  Figure 6 presents an example of this stage.

### 2.3. Classification of the Features

Classifiers typically used in most of the procedures for analyzing medical images can be divided into the following categories: conventional classifiers, artificial neural networks [3, 6, 38, 39], fuzzy systems [40], and support vector machines [41, 42]. A key aspect to consider is the problem known as the* curse of dimensionality*: a classifier with a high dimensionality requires a large number of training samples to avoid overfitting. However, in our case, the number of available samples is very reduced, so simple classifiers based on distances between histograms are applied.

In particular, the Bhattacharyya distance [42] is used to provide a measure of the similarity of two histograms. Considering two histograms *H*_1_ and *H*_2_, this distance is defined by

(3) $$\begin{array}{c} d\left(\;H_{1},H_{2}\;\right)=\sqrt{1-\overset{255}{\underset{i=0}{\sum }}\sqrt{H_{1}\left(i\right)\cdot H_{2}\left(i\right)}}. \end{array}$$

Let us assume a training set of *n* radiographs, *T* = {*T*_1_, *T*_2_,…, *T*_*n*_}, and a new radiograph *I* to classify. The 24 LBP histograms of all the images are computed (both training set and *I*). Then, each histogram of *I* is compared with *n* corresponding histograms in *T* using (3). After that, the difference between the minimum distance to the normal radiographs of *T* and the minimum distance to the pathological radiographs of *T* is computed. That is, the system calculates for each region *R* in image *I*:

(4) vR=mino∈normal⁡ dHRI,HRTo−minp∈pathologic⁡ dHRI,HRTp,

where normal is the set of normal radiographies in *T* and pathologic is the set of pathologic ones. The values *v*(*R*) can be interpreted as* votes* to either normality or pathology; a high negative value should be obtained for regions similar to the normal samples and a high positive value for the nonnormal samples. Therefore, the set of 24 values, {*v*(1), *v*(2),…, *v*(24)}, provides information that has to be combined in a final classification. Three different approaches are proposed for this purpose:

(1) *GDAV: Greater Difference in Absolute Value*. This technique consists of obtaining the maximum value of |*v*(*R*)| for all *R* in {1,2,…, 24}. If the corresponding *v*(*R*) is a negative number, then image *I* is classified as normal; otherwise, it is classified as pathologic. This method considers that the region which has a greater difference is the one that contains most information for the problem.
(2) *DV: Discrete Voting*. In this case, all regions contribute to the final classification. The sign of each *v*(*R*) is considered as a vote to normality (negative sign) or to pathology (positive sign). The class with the most votes provides the final classification for *I*.
(3) *CV: Continuous Voting*. A potential drawback of DV method is that relevant information can be lost when discretizing the values of *v*(*R*). To avoid this problem, CV takes the sum *v*(1) + *v*(2)+⋯+*v*(24). If the sum is positive, image *I* is considered as pathologic and otherwise normal. In fact, the optimum decision threshold is not necessarily 0, but it can be slightly biased. This threshold determines the compromise between false positive and false negative errors.

These three classification techniques assume that all the regions of the images have the same information for the problem. However, this could not be the case if some areas are more discriminant than others. Therefore, we have studied the use of matrix that weighs the relative importance of each region of interest. It is called* discrimination matrix* and can be defined as a function *w*(*R*) of real values from 0 to 1, for each *R* in {1,2,…, 24}. These weights are obtained from the same set of training data. When using the discrimination matrix in classification, all the *v*(*R*) are substituted by the product *v*(*R*)*w*(*R*). The three classifiers described above are evaluated both using the weighted values and not using them.

## 3. Results and Discussion

The set of 48 digital chest radiographs (25 normal and 23 pathologic) described in Section 2 has been used in the experimental validation of the proposed method. The testing procedure performs a leave-one-out process, which consists in removing one image from the data set, *I*, and takes the rest of images as the training set, *T*. Image *I* is classified against *T* using LBP histograms and the 6 classifiers described above (GDAV, DV, and CV; using discrimination matrix or not). If the predicted class is different from the real class of *I*, then there is a classification error. This process is repeated for all the available images. The* success rate* of a classifier is defined as the number of correctly classified images with respect to the total number of images.

### 3.1. Experimental Results

The success rates obtained for all the classifiers in the validation experiments of the technique are presented in Table 1. These results are indicated for males, females, and using all individuals.

**Figure 7:**
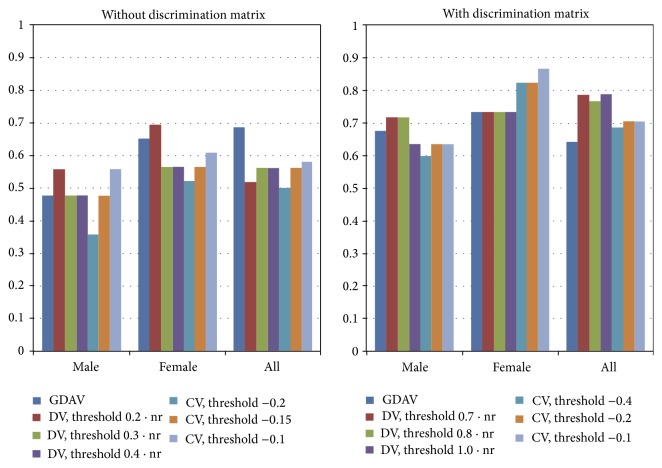
Success rates of the classifiers with and without discrimination matrix and using different thresholds. nr represents the number of existing radiographs in the training set.

We were also interested in studying the effect of the threshold in the voting methods.  Figure 7 shows a graphical comparison of the three classifiers, with and without weighting matrix, using different thresholds for DV and CV methods.

### 3.2. Discussion of the Results

In a problem of binary classification, as the present one, the expected error rate of a completely random classifier would be 50%. The fact that some experiments, for example, GDAV method in the male subset, produce a higher error is an evidence of the complexity of the tackled problem. Besides the implicit difficulty of the problem, the small number of images available poses an additional challenge. To get a sample of all possible variations of sex, age, pathologies, and so forth, some thousands of radiographs would be necessary. For example, some classifiers in Table 1 produce better results with the complete set than with only the male/female set.

There is not a method clearly yielding the best accuracy for all the tests, although voting schemes, DV and CV, usually obtain less error rates.  Figure 7 shows that the correct selection of the threshold, specially in CV method, can affect greatly its effectiveness. In CV, the optimum threshold appears to be near 0, as it would be expected.

Regarding the comparison between using or not the discrimination matrix, there is very strong evidence that using it has a big benefit in the obtained results. Almost all methods achieve a significant improvement applying the matrix of weights, with an average of 21% higher accuracy. The best result is obtained with CV method, giving 87% of correct classifications. Considering the set of all images, the optimum classifier is DV with discrimination matrix, producing 79% of accuracy.

A relevant limitation of our data set is that the areas of abnormality are not marked in the available pathological images; indeed, these radiographs may contain many normal regions. This fact hinders the distinction of normality. The improvement achieved by using the discrimination matrix shows that this problem has a great effect in the results. A greater benefit could be obtained if pathological areas were precisely marked in the training samples.

### 3.3. Hypothesis Testing

In this subsection, information is provided on the procedure followed to conduct an experiment to investigate if the proportion of cases which were correctly classified is the same for all the classifiers (GDAV, DV, and CV). The following hypotheses are proposed in this study:

(H0) * Null Hypothesis*. The classifiers GDAV, DV, and CV are equally effective.
(H1) * Alternative Hypothesis*. There is a difference in effectiveness among the classifiers GDAV, DV, and CV.

The metric selected to measure classifiers effectiveness was correct detection (normality/pathology). Thus, classifier effectiveness is the dependent variable, and the kind of classifier is the independent variable. Since the dependent variable is dichotomous, Cochran's *Q* nonparametric statistical test is employed to verify if the three classifiers have identical effects.

Table 2 shows the results obtained using SPSS 19.0 statistical software package. Based on the observed significance levels, we can reject the null hypothesis; that is, there are statistically significant differences with regard to the correct detections among the classifiers, for the set* All χ* ^2^(2) = 5.28 (*p* = 0.07) with discrimination matrix. However, Cochran's *Q* test did not indicate any differences among the three classifiers (value higher than 0.1) in all the other cases.

## 4. Conclusions

A new approach for detecting normality/pathology in chest radiographies has been presented. Our method is based on LBP as a simple but powerful texture descriptor. LBP histograms of different lung regions are classified and then combined to produce the final classification. Different combination schemes have been compared, and a statistical analysis has found that in some cases there is a significant difference among them.

In general, the use of a discrimination matrix yields an average improvement around 20% in the success rates. This fact indicates that not all regions have the same importance in the pathology detection. Moreover, when a pathology is detected, the obtained distances could be used to identify the regions with most probability of abnormality. This can be helpful to the medical professionals, which can center their attention in the suspicious areas.

The obtained success rate is near 90% for the best classifiers. There is clearly an important margin for improvement, since the developed system is a prototype for research purposes. In order to be introduced in the context of a hospital, a better accuracy would be required. One disadvantage of the proposed approach is that it requires a large number of images, in order to have enough samples of all appearances for different sex, age, kinds of pathologies, and so forth. Therefore, a large set of chest radiographs would be needed to improve the results. Moreover, as discussed above, these images should be marked more precisely with the areas of pathology. Another limitation of the method is that it relies only on texture information. Some types of diseases, for example, affecting only the intensity of the images, would not be detected. To overcome this issue, the combination of other image descriptors could be applied, such as scale-invariant features or deep learning methods. In any case, we have to recall that these kinds of systems are designed to help radiologists, not to replace them.

## Figures and Tables

**Table 1:** Success rates (as a percentage from 0 to 1) of classification using GDAV, DV, and CV methods, with and without discrimination matrix. The best result for each classifier is marked in bold. In the male/female tests, only those classes are included in the training and testing process.

	Without discr. matrix			With discr. matrix		
	Male	Female	All	Male	Female	All
GDAV	0.48	0.65	0.69	0.68	**0.74**	0.65
DV	0.56	0.69	0.56	0.72	0.74	**0.79**
CV	0.56	0.61	0.56	0.64	**0.87**	0.71

**Table 2:** Results of the hypothesis testing on the GDAV, DV, and CV classifiers, without and with discrimination matrix, and using male, female, and all radiographs. *N* is the sample size, and df means degrees of freedom.

	Without discr. matrix			With discr. matrix		
	Male	Female	All	Male	Female	All
*N*	25	23	48	25	23	48
Cochran's *Q*	1.14	0.75	2.05	0.46	2.57	5.28
df	2	2	2	2	2	2
Asymptotic significance	0.56	0.68	0.35	0.79	0.27	0.07
